# Association between perceived greenness and physical exercise among university students: nature connectedness as a statistical mediator and gender as a moderator

**DOI:** 10.3389/fpubh.2026.1978168

**Published:** 2026-09-29

**Authors:** Hongxiang Jia, Yincheng Liu

**Affiliations:** Department of Physical Education, Tianjin University of Commerce, Tianjin, China

**Keywords:** gender, nature connectedness, perceived greenness, physical exercise, public mental health, social-ecological model, statistical mediation, university students

## Abstract

**Background:**

Supportive residential environments and regular physical exercise may serve as upstream resources for health promotion among university students. However, whether perceived greenness is associated with exercise through nature connectedness, and whether this pattern differs by gender, remains unclear.

**Methods:**

We analyzed cross-sectional data from 3,418 university students in China in the cleaned EPOCH-A dataset. Perceived greenness of the longest-lived residential environment was assessed using neighborhood and window-view greenness items. Nature connectedness was measured using the single-item Inclusion of Nature in Self scale, and physical exercise during the previous month using the Physical Activity Rating Scale-3. Adjusting for age, subjective socioeconomic status, grade, and residential setting, we estimated a conditional process model equivalent to PROCESS Model 14, using HC3 robust standard errors and 5,000 bootstrap resamples.

**Results:**

Perceived greenness was positively correlated with nature connectedness (r = 0.288, *p* < 0.001) and physical exercise (*r* = 0.068, *p* < 0.001), while nature connectedness was positively correlated with exercise (*r* = 0.131, *p* < 0.001). After covariate adjustment, perceived greenness was positively associated with nature connectedness (*B* = 0.241, 95% CI 0.211–0.271). The nature connectedness-by-gender interaction was significant (*B* = −1.953, 95% CI −2.885 to −1.022), with a stronger nature connectedness–exercise association in men (*B* = 2.614) than women (*B* = 0.660). The conditional indirect association of perceived greenness with exercise through nature connectedness was 0.630 (95% bootstrap CI 0.424–0.853) in men and 0.159 (95% bootstrap CI 0.052–0.270) in women. The index of moderated mediation was −0.471 (95% bootstrap CI −0.705 to −0.251). Findings were directionally consistent across alternative continuous exercise measures.

**Conclusions:**

Perceived greenness, nature connectedness, and physical exercise showed modest but consistent concurrent associations, with a stronger nature connectedness–exercise association in men. Because the study was cross-sectional and exposure and outcome had different temporal reference frames, causal mediation or temporal ordering cannot be established.

## 1 Introduction

University students are navigating rapid changes in education, residential contexts, digital life, and urban environments at a developmental stage already marked by substantial mental-health vulnerability. Public mental health therefore increasingly considers upstream protective resources alongside clinical services, including modifiable health behaviors and supportive community environments. Physical activity is one such resource: systematic reviews and meta-analyses indicate that physical-activity interventions can reduce symptoms of anxiety, depression, and stress in undergraduate populations, although effects vary across intervention types and study quality (1–4). At the same time, university students often face time, motivational, social, and environmental constraints on maintaining regular activity (5–7). A social-ecological perspective is useful in this context because it treats health behavior as embedded in interacting individual, social, organizational, and environmental conditions rather than as a product of motivation alone (8). Recent work further highlights that environmental factors such as extreme heat exposure are associated with depressive symptoms in adolescent populations (9), and that digital literacy and social media communication can shape sports participation attitudes and physical activity behavior (10).

Greenness is a potentially supportive environmental resource. Conceptual models of nature and health propose that green environments may be related to health through reduced harmful exposures, psychological restoration, opportunities for physical activity, and social interaction (11–13). Evidence on physical activity generally points to positive but often small and heterogeneous associations with greenspace exposure, depending on how greenness, spatial scale, and activity are measured (14, 15). Research in higher-education settings likewise suggests that walkability, perceived naturalness, green space, and accessible outdoor areas are associated with student activity or health, while also emphasizing the predominance of cross-sectional and self-reported evidence (16–19). From an upstream public mental health perspective, such environments may matter partly because they shape access to health-promoting behaviors that are themselves associated with psychological wellbeing (1, 2).

Two influential theoretical frameworks inform why green environments may be related to health and health-promoting behavior. Attention Restoration Theory (ART) (20) proposes that natural environments restore directed attention capacity through “soft fascination”—effortless attention that allows recovery from mental fatigue—and that this restoration may in turn support engagement in health-promoting behaviors such as physical activity. Stress Reduction Theory (SRT) (21) posits that natural settings evoke positive affective responses and reduce physiological stress arousal, which may facilitate activity participation. Both theories suggest that the psychological benefits of green environments may operate through restoration and stress reduction pathways. Nature connectedness may represent an individual difference that moderates the extent to which people notice, engage with, and benefit from natural environments, potentially strengthening the link between environmental perception and health behavior.

Objective environmental quantity and perceived environmental experience are not interchangeable. Visibility, perceived quality, comfort, and subjective naturalness may have behavioral and psychological relevance beyond mapped greenspace alone. Previous studies have assessed perceived neighborhood greenness, green views from home, and access to nearby green space (22), and reviews of university environments show that subjective and objective indicators do not always have identical associations with student outcomes (16, 18). In the present questionnaire, perceived greenness referred to the place where participants had lived for the longest period of their lives and was assessed using neighborhood greenness and greenness visible from home. The exposure therefore represents perceived greenness of a long-term residential environment rather than current campus greenness.

Nature connectedness may represent a psychosocial correlate of how environmental resources are experienced. It refers to the extent to which people experience a cognitive, affective, or experiential relationship with the natural world (23, 24). Schultz's Inclusion of Nature in Self framework represents this relationship as increasing overlap between circles denoting the self and nature, and later work expanded the measurement of related constructs (23, 25, 26). Reviews and meta-analyses indicate that nature contact, positive experiences in nature, and deliberate engagement with nature are associated with greater nature connectedness (27, 28). Nature connectedness is also associated, generally at small-to-moderate magnitudes, with happiness, eudaimonic wellbeing, and other positive psychological outcomes (29–31). These findings suggest that perceived greenness and nature connectedness may capture complementary environmental and psychosocial resources relevant to mental-health promotion, even when mental health is not measured directly.

Evidence connecting nature connectedness with physical activity is less consistent. Among regular exercisers, nature relatedness is associated with activity environment and psychological experiences of exercise (32), and greater nature connectedness has been associated with more nature-based recreation among students (33). In contrast, Teixeira et al. (34) found that nature connectedness did not explain variation in overall physical activity among adults and older adults. More recently, a three-wave study of Chinese university students reported that nature relatedness prospectively predicted greater nature-based physical activity at the subsequent wave, which in turn was associated with later positive mental health, although the effects were small (35). These findings raise the possibility that nature connectedness is associated with the behavioral use of supportive environmental resources, but its relationship with overall exercise remains uncertain. These findings align with broader evidence that physical activity is associated with a range of psychosocial outcomes through mediation and moderation processes (36, 37).

Equity is also relevant because environmental and behavioral resources may not translate into health-promoting activity equally across groups. A study of college students in Brazil and the United States found that women reported greater connection to nature and stronger outdoor preferences but participated less in nature-based recreation than men (38). Reviews of physical-activity barriers in university populations identify time, facilities, social support, and motivation as common constraints, while research focused on young women additionally highlights safety, body image, social evaluation, and gendered norms (5, 6, 39, 40). Broader evidence also documents persistent gender inequalities in physical activity participation. Thus, similar levels of nature connectedness may not correspond to equally strong exercise associations across gender groups, making gender a potentially important marker of differential access to or use of protective resources. Health communication through sports-related media has also been shown to shape sport participation attitudes differently across groups (41).

Drawing on social-ecological and nature-connectedness perspectives, we examined concurrent statistical associations among perceived greenness of long-term residential environments, nature connectedness, and physical exercise in university students, and tested whether gender moderated the nature connectedness–exercise association. This design addresses two themes central to upstream public mental health research: supportive environmental resources and group differences in the extent to which such resources are linked with health-promoting behavior. Because the study is cross-sectional, the exposure and outcome variables refer to different time windows (long-term residence vs. previous month), and the study does not include a direct mental-health outcome, the hypotheses concern statistical associations rather than causal effects or demonstrated mental-health benefits: H1, perceived greenness is positively associated with physical exercise; H2, perceived greenness has a significant statistical indirect association with physical exercise through nature connectedness; H3, the positive association between nature connectedness and physical exercise is stronger in men; and H4, the conditional statistical indirect association is larger in men than in women (Figure 1).

**Figure 1:**
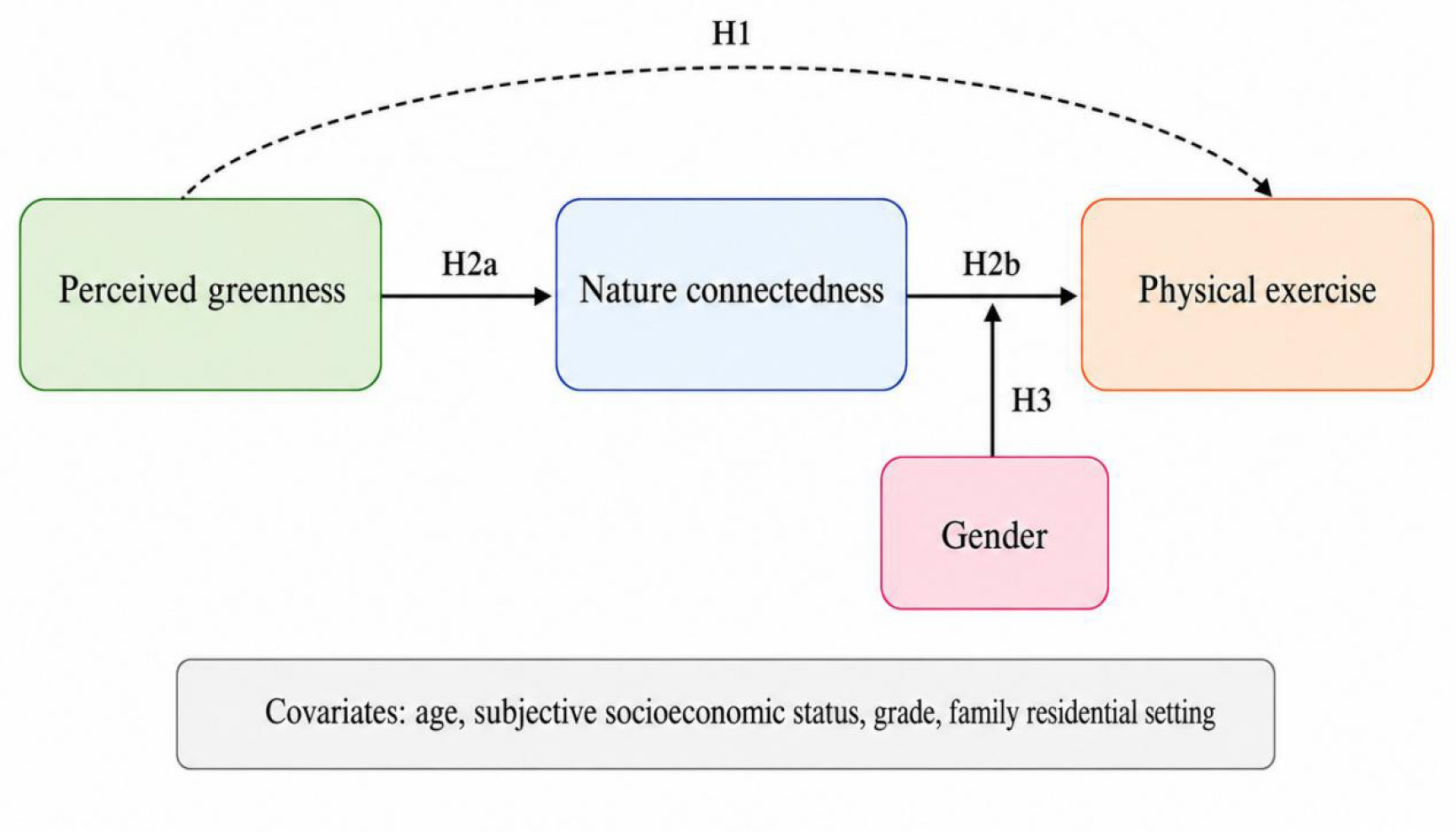
Hypothesized model of the associations among perceived greenness, nature connectedness, physical exercise, and gender moderation.

## 2 Materials and methods

### 2.1 Study design and participants

This study was a cross-sectional secondary analysis of the EPOCH-A digital behavior and environmental psychology survey, administered online to university students in China in 2024. The archived project materials describe the dataset as a nationwide cross-sectional sample; however, a site-by-site recruitment log was not retained in the files available for this analysis, and the number recruited through each institution, region, or distribution channel could not be reconstructed. We therefore refer to the sample as a multi-site online sample of university students in China and do not claim national representativeness. Before beginning the survey, participants read an electronic informed-consent statement explaining that participation was voluntary, personal information would be kept confidential, and they could withdraw at any time.

We used the project's cleaned dataset. The survey and data-quality design incorporated checks for questionnaire completeness, missing core variables, clearly anomalous or internally inconsistent response patterns, and an attention-check item. The archived materials available for this secondary analysis did not contain a case-by-case exclusion log; therefore, the original number approached, the number removed at each cleaning step, and the response rate could not be reconstructed. The cleaned dataset contained 3,418 participants, all of whom had complete data on the core study variables and were included in the analysis.

The sample included 1,348 men (39.4%) and 2,070 women (60.6%), aged 15–25 years (mean 18.56, SD 1.05). There were 2,202 first-year students (64.4%), 654 second-year students (19.1%), 515 third-year students (15.1%), 44 fourth-year students (1.3%), one post-graduate student, and two students in other categories. Because the upper-year groups were small, fourth-year, post-graduate, and other students were combined as “fourth year or above” (*n* = 47, 1.4%) in regression analyses. Family residential settings were urban for 1,004 participants (29.4%), suburban/peri-urban for 765 (22.4%), and rural for 1,649 (48.2%).

The study protocol was approved by Academic Ethics Committee of Shaanxi Police College (approval no. 20240721-001). All participants provided electronic informed consent before completing the survey.

### 2.2 Measures

#### 2.2.1 Perceived greenness

Perceived greenness was assessed with two items: “the level of greenness in the community where you lived” and “the green view seen from the window of your home.” The questionnaire was administered in Chinese, the participants' native language. The English translations provided here were prepared through forward translation by two bilingual researchers with expertise in environmental psychology and physical education, followed by back-translation by a third bilingual researcher and consensus review to ensure semantic equivalence. The measurement approach is consistent with perceived-environment studies that assess subjective green exposure in everyday residential settings (22). Each item was rated on a five-point scale from 1 (no greenness) to 5 (very high greenness). The questionnaire instructed participants to answer these environmental items with reference to the place where they had lived for the longest period of their lives; we therefore refer to the construct as perceived greenness of the long-term residential environment. This brief measure captures subjective perception of residential greenness visibility and does not assess accessibility, quality, safety, or usability of green space. The two items were summed (range 2–10), with higher scores indicating greater perceived greenness. In the present sample, the inter-item correlation was *r* = 0.800 and Cronbach's alpha was 0.888, supporting their combination as a brief perceived-greenness index. Analyses for each item separately yielded directionally consistent results: neighborhood greenness correlated *r* = 0.261 with nature connectedness and *r* = 0.057 with exercise (both *p* < 0.001); window-view greenness correlated *r* = 0.266 with nature connectedness and *r* = 0.058 with exercise (both *p* < 0.001). The inter-item correlation was *r* = 0.811.

#### 2.2.2 Nature connectedness

Nature connectedness was measured using Schultz's single-item Inclusion of Nature in Self (INS) measure (23). Participants selected one of seven pictorial options (A–G) showing progressively greater overlap between circles representing “self” and “nature.” Related pictorial extensions have subsequently been used to assess human–nature connection (25, 26). Responses were coded from 1 to 7 according to increasing overlap, with higher scores indicating stronger nature connectedness.

#### 2.2.3 Physical exercise

Physical exercise during the previous month was assessed using the three core components of the Physical Activity Rating Scale-3 (PARS-3): exercise intensity, duration per session, and frequency. PARS-3 has a long history of use in research on Chinese university students (42). Intensity and frequency were scored from 1 to 5, and duration was scored from 0 to 4. Because duration was stored in the dataset using raw codes 1–5, the exercise score was calculated as intensity × (raw duration code – 1) × frequency, yielding a range of 0–100. Higher scores indicate greater exercise volume. For descriptive and sensitivity analyses, scores ≤ 19 were classified as low, 20–42 as moderate, and ≥43 as high exercise volume. The continuous score was used in the primary model.

#### 2.2.4 Covariates

Covariates were age, subjective socioeconomic status, academic grade, and family residential setting. Subjective socioeconomic status was assessed using a single 1–10 self-rating modeled on the logic of the MacArthur subjective social-status measure (43), with higher scores indicating higher perceived socioeconomic standing. Grade and residential setting were dummy coded. In the cleaned dataset, the gender variable contained only male and female responses; men were coded 0 and women 1 for the moderation analysis. Interpretation of this variable is limited to group differences represented by this binary survey item.

### 2.3 Statistical analysis

Analyses were conducted in Python using statsmodels. Means, standard deviations, and Pearson correlation coefficients were first calculated. Because the PARS-3 score was markedly right-skewed, Spearman rank correlations were also reported as a distribution-robust descriptive check.

Conditional process analysis followed a regression specification equivalent to Hayes PROCESS Model 14 (44): perceived greenness was X, nature connectedness *M*, physical exercise Y, and gender W, with W moderating the M → Y path. Perceived greenness and nature connectedness were mean centered before construction of the interaction term. Primary models adjusted for age, subjective socioeconomic status, grade, and residential setting. The Breusch–Pagan test indicated heteroskedasticity in the exercise outcome model (*p* < 0.001), so HC3 heteroskedasticity-robust standard errors are reported. Statistical indirect effects, conditional indirect effects, and the index of moderated mediation were estimated using 5,000 non-parametric bootstrap resamples; 95% bootstrap confidence intervals not containing zero were considered statistically significant.

Because all focal variables were measured cross-sectionally and refer to different time windows (long-term residential environment vs. previous-month exercise), indirect-effect estimates are described as statistical indirect effects or conditional indirect associations rather than causal mediation. Cross-sectional mediation parameters can differ systematically from the longitudinal process they are intended to represent (45, 46), and the estimates should be interpreted as reflecting a covariance structure at a single point in time rather than temporal ordering.

Sensitivity analyses repeated the conditional process model using three alternative continuous exercise scores: (1) the original database sum of intensity, duration, and frequency (range 3–15); (2) ln(1+PARS-3); and (5) the mean of standardized intensity, duration, and frequency items. We additionally fitted an ordinal logistic model using low/moderate/high PARS-3 categories and a binary logistic model comparing low vs. moderate/high exercise. Finally, walking distance to the nearest park or green space was added as an additional covariate. All tests were two sided with α = 0.05.

## 3 Results

### 3.1 Descriptive statistics and correlations

Mean perceived greenness was 7.64 (SD 1.84), mean nature connectedness was 4.16 (SD 1.62), and mean PARS-3 exercise score was 14.52 (SD 20.15). The median PARS-3 score was 6 (interquartile range 2–18; range 0–100). Scores were markedly right-skewed (skewness = 2.21), and 651 participants (19.0%) scored 0. Using conventional categories, 2,646 participants (77.4%) were in the low-exercise group, 425 (12.4%) in the moderate group, and 347 (10.2%) in the high group. Gender-stratified descriptive statistics showed that women reported slightly higher perceived greenness (*M* = 7.69, SD = 1.80) and nature connectedness (*M* = 4.14, SD = 1.62) than men (perceived greenness *M* = 7.45, SD = 1.91; nature connectedness *M* = 4.12, SD = 1.69), but substantially lower exercise volume (women *M* = 9.95, SD = 14.93 vs. men *M* = 21.06, SD = 24.72).

Pearson correlations showed that perceived greenness was positively associated with nature connectedness (*r* = 0.288, *p* < 0.001) and weakly with physical exercise (*r* = 0.068, *p* < 0.001); the latter correlation was small in magnitude. Nature connectedness was also positively associated with exercise (*r* = 0.131, *p* < 0.001). Corresponding Spearman correlations were 0.285, 0.074, and 0.193, respectively; all were *p* < 0.001 (Table 1).

**Table 1:** Descriptive statistics and Pearson correlations among study variables (*N* = 3,418).

Variable	*n*	M/%	SD
Age	3,418	18.56	1.05
Gender (female)	3,418	60.6%	—
Grade (year 1)	3,418	64.4%	—
Residence (rural)	3,418	48.2%	—
Subjective SES	3,418	—	—
Perceived greenness	3,418	7.64	1.84
Nature connectedness	3,418	4.16	1.62
Physical exercise (PARS-3)	3,418	14.52	20.15
Correlations	1	2	3
1. Perceived greenness	1		
2. Nature connectedness	0.288^***^	1	
3. Physical exercise	0.068^***^	0.131^***^	1

Gender coded 0 = male, 1 = female. Grade coded as year 1 = 1, year 2 = 2, year 3 = 3, year 4+ = 4. Residence coded 1 = urban, 2 = suburban, 3 = rural. For categorical variables, the M/% column reports the percentage of the modal category (female, year 1, rural, respectively). Perceived greenness is the sum of two 5-point items (range 2–10). Subjective SES was assessed on a 1–10 scale; M/SD not reported in original cleaning. ^***^p < 0.001.

### 3.2 Regression associations

After adjustment for age, subjective socioeconomic status, grade, and residential setting, perceived greenness was positively associated with nature connectedness (*B* = 0.241, β = 0.266, SE = 0.015, 95% CI 0.211–0.271, *p* < 0.001); the nature-connectedness model explained 9.7% of the variance (*R*^2^ = 0.097).

In an outcome model excluding nature connectedness and the interaction term, the total association between perceived greenness and exercise was *B* = 0.756 (β = 0.070, SE = 0.196, 95% CI 0.371–1.140, *p* < 0.001). In the full model, the direct association between perceived greenness and exercise remained significant but was small in magnitude (*B* = 0.521, β = 0.041, HC3 SE = 0.196, 95% CI 0.138–0.905, *p* = 0.008). The nature connectedness-by-gender interaction was significant (*B* = −1.953, β = −0.174, HC3 SE = 0.475, 95% CI −2.885 to −1.022, *p* < 0.001). The exercise model had *R*^2^ = 0.111 and adjusted *R*^2^ = 0.108; the interaction contributed approximately Δ*R*^2^ = 0.006, indicating a small increment in explained variance, with the majority of variance explained by covariates and gender rather than the focal environmental variables (Table 2).

**Table 2:** Main regression results from the conditional indirect-effect model.

Predictor	Outcome	*B*	HC3 SE	95% CI	*p*	β
Perceived greenness	Nature connectedness	0.241	0.015	0.211 to 0.271	< 0.001	0.266
Perceived greenness	Physical exercise	0.521	0.196	0.138 to 0.905	0.008	0.041
Nature connectedness	Physical exercise	2.614	0.429	1.773 to 3.454	< 0.001	0.233
Gender (women = 1)	Physical exercise	−11.753	0.757	−13.236 to −10.270	< 0.001	—
Nature connectedness × gender	Physical exercise	v1.953	0.475	−2.885 to −1.022	< 0.001	−0.174

B, unstandardized regression coefficient; β, standardized regression coefficient; HC3 SE, heteroscedasticity-consistent standard error (HC3 estimator); 95% CI, 95% confidence interval. Models adjusted for age, subjective socioeconomic status, grade, and residential setting. Perceived greenness is the sum of two 5-point items (range 2–10). Gender is coded 0 = male, 1 = female; the nature connectedness coefficient represents the simple slope at gender = 0 (male).

Index of moderated mediation = −0.471; bootstrap 95% CI −0.705 to −0.251.

### 3.3 Gender moderation and conditional indirect associations

Simple-slope analysis showed a positive association between nature connectedness and exercise among men (*B* = 2.614, SE = 0.429, 95% CI 1.773–3.455, *p* < 0.001). The association was also positive among women but smaller in magnitude (*B* = 0.660, SE = 0.226, 95% CI 0.217–1.103, *p* = 0.004).

The conditional indirect association of perceived greenness with exercise through nature connectedness was positive and statistically significant among men (indirect effect = 0.630, Bootstrap 95% CI 0.424 to 0.853) and among women (indirect effect = 0.159, Bootstrap 95% CI 0.052 to 0.270), but was substantially larger among men. The index of moderated mediation was negative and statistically significant (index = −0.471, Bootstrap 95% CI −0.705 to −0.251), confirming that gender moderated the indirect pathway.

### 3.4 Sensitivity analyses

The pattern was consistent across alternative continuous exercise scores. Using the original 3–15 sum score, the nature connectedness-by-gender interaction was *B* = −0.259 (*p* < 0.001) and the index of moderated mediation was −0.062 (95% bootstrap CI −0.094 to −0.033). Using ln(1+PARS-3), the interaction was *B* = −0.098 (*p*≈0.001) and the index was −0.024 (95% bootstrap CI −0.037 to −0.010). Using the mean of standardized exercise components, the interaction was *B* = −0.071 (*p* < 0.001) and the index was −0.017 (95% bootstrap CI −0.026 to −0.009; Table 3). The PARS-3 score correlated strongly with the original 3–15 sum score (Pearson *r* = 0.883; Spearman ρ = 0.964).

**Table 3:** Sensitivity analyses using alternative continuous physical-exercise scores.

Exercise outcome	NC × gender *B*	Interaction *p*	Index of moderated mediation	Bootstrap 95% CI
PARS-3 standard score	−1.953	< 0.001	−0.471	−0.705 to −0.251
Original 3–15 sum score	−0.259	< 0.001	−0.062	−0.094 to −0.033
ln(1+PARS-3)	−0.098	≈0.001	−0.024	−0.037 to −0.010
Mean standardized component score	−0.071	< 0.001	−0.017	−0.026 to −0.009

When PARS-3 was categorized as low, moderate, or high, perceived greenness (OR = 1.078, *p* = 0.002) and nature connectedness (OR = 1.201, *p* < 0.001) were associated with higher exercise category, but the nature connectedness-by-gender interaction did not reach the conventional *p* < 0.05 threshold (OR = 0.910, *p* = 0.071). A binary low-vs.-moderate/high model yielded a similar interaction result (OR = 0.916, *p* = 0.101). These findings qualify the gender moderation result: the interaction is robust for continuous exercise volume but does not reliably predict categorical exercise thresholds. After additional adjustment for walking distance to the nearest park or green space, the perceived greenness–nature connectedness coefficient was *B* = 0.239, the interaction remained significant (*B* = −1.901, *p* < 0.001), and the index of moderated mediation was approximately −0.454 (95% bootstrap CI −0.687 to −0.234). Variance inflation factors for the main predictors ranged from approximately 1.06 to 2.60. As an additional sensitivity analysis addressing the temporal reference mismatch, we stratified the primary model by current residential setting (urban, suburban/peri-urban, rural). The nature connectedness-by-gender interaction remained directionally consistent and statistically significant across all three groups (urban: *B* = −2.692, *p* < 0.001; suburban: *B* = −2.651, *p* < 0.001; rural: *B* = −1.924, *p* < 0.001), although the dataset did not contain information on current campus residence or on-campus vs. off-campus accommodation that would allow a more direct test of the temporal mismatch.

## 4 Discussion

### 4.1 Perceived greenness and physical exercise as linked protective resources

Perceived greenness of the long-term residential environment was positively, but weakly, associated with exercise during the previous month, and the association remained after adjustment for demographic covariates. The exposure and outcome variables refer to different time windows: perceived greenness was assessed for the place where participants had lived for the longest period, potentially spanning many years, whereas exercise was assessed for the previous month. These two measures may therefore not refer to the same residential environment or the same time period, and the observed association should be interpreted as a concurrent statistical association rather than a temporally ordered effect. The direction of the association is, however, consistent with the broader literature on greenspace and physical activity. Social-ecological models position the physical environment as one component of activity behavior (8), while green environments may be related to physical activity by providing places for walking and recreation, increasing environmental attractiveness, and supporting routine outdoor activity (11–14). Recent systematic reviews and meta-analyses likewise suggest that associations between greenness and physical activity are generally small and heterogeneous (14, 15).

The small effect size is compatible with the multifactorial nature of exercise among university students. Reviews identify time constraints, activity goals, social support, facilities, and environmental resources as concurrent influences (5–7). The present findings therefore do not support treating perceived greenness as a dominant determinant of exercise. From an intervention perspective, the observed environmental association is small (*r* = 0.068 for the greenness–exercise correlation, and ΔR^2^ = 0.006 for the gender interaction), and changes in perceived greenness alone would be unlikely to produce meaningful increases in exercise at the individual level. At a population level, however, even small environmental associations may be relevant when distributed across large groups, particularly when combined with other intervention strategies. Their relevance to public mental health is upstream rather than clinical: physical activity is a modifiable behavior associated with lower anxiety, depression, and stress in university populations, and recent syntheses support its use as one component of mental-health promotion (1, 2). Our study did not measure those outcomes, so it cannot show that the observed greenness–exercise association translated into better mental health. It does, however, identify a small statistical association between a supportive environmental characteristic and a behavior with established mental-health relevance. Digital literacy and media environment also play roles in shaping sports participation behavior, particularly in rural and underserved populations (10).

### 4.2 Nature connectedness as a psychosocial correlate of environmental resources

The association between perceived greenness and nature connectedness was considerably stronger than the direct correlation between perceived greenness and exercise. Theory and empirical work identify nature contact, positive experiences in nature, and deliberate engagement with nature as important correlates or antecedents of nature connectedness (23, 27, 28). Long-term residence in greener environments may coincide with more frequent or meaningful nature experiences; however, we did not measure accumulated nature contact and cannot determine whether perceived greenness contributes to nature connectedness or whether highly nature-connected individuals attend more to and evaluate surrounding greenness more favorably.

The positive association between nature connectedness and exercise is broadly consistent with findings from nature-based recreation and green-exercise research (32, 33, 47, 48) and with the recent longitudinal finding that nature relatedness prospectively predicted nature-based physical activity among Chinese university students (35). At the same time, null findings have been reported for overall physical activity in other age groups (34). Because our outcome captured overall exercise rather than specifically nature-based activity, the present analysis suggests that psychological connection with nature may covary with broader exercise behavior. This is relevant to the Research Topic's focus on protective resources because it points to a psychosocial dimension of environmental support: the same physical environment may be perceived and behaviorally used differently depending on an individual's relationship with nature. The observed associations were modest, and future studies should distinguish indoor exercise, ordinary outdoor activity, and activity undertaken in natural settings.

The bootstrap analysis identified a significant statistical indirect association from perceived greenness to exercise through nature connectedness. This result supports the inclusion of human–nature psychological relationships in analyses of supportive environments and health-promoting behavior. However, it should not be interpreted as evidence that nature connectedness causally mediates an effect of greenness on exercise. Methodological work shows that cross-sectional mediation estimates can be systematically biased relative to longitudinal processes (45, 46), particularly when variables refer to different time periods as in the present study. The current result therefore indicates that the observed covariance structure is consistent with the hypothesized indirect statistical pathway, while temporal and causal ordering remain untested. Future longitudinal research with at least three measurement waves is needed to establish whether nature connectedness temporally mediates the greenness–exercise relationship.

### 4.3 Gender differences and equity

Nature connectedness was positively associated with exercise in both men and women, but the slope and conditional indirect association were larger in men. Rosa et al. (38) reported that female college students in Brazil and the United States showed stronger connection to nature and outdoor preferences yet lower participation in nature-based recreation than male students. This pattern is compatible with our observation that psychological connection with nature and activity behavior need not covary equally strongly across gender groups. In an equity-oriented interpretation, the finding concerns differential translation of a potentially protective psychosocial resource into a health-promoting behavior rather than a difference in the value of nature connectedness itself.

Research on barriers to physical activity provides plausible contextual hypotheses for the observed gender difference, although these mechanisms were not measured in the present study. Common barriers among university students include time, facilities, social support, and motivation (5, 6), while work focused on young women additionally highlights safety, body image, social evaluation, and gendered norms (39, 40). Such constraints could weaken the correspondence between affinity with nature and actual exercise for some groups. The observed interaction may also reflect gender differences in exercise type, baseline activity levels, residential experience, or unmeasured socioeconomic and cultural factors. These explanations are drawn from external literature and should be treated as hypotheses for future research rather than as findings of this study. The moderation result should not be interpreted as evidence of fixed biological differences between men and women. The cleaned dataset contained only male and female categories, so the analysis also cannot represent gender-diverse students.

The interaction remained significant across several continuous exercise scores but fell below the conventional 0.05 threshold when PARS-3 was reduced to low, moderate, and high categories. This qualifies the equity-related finding: the data support gender heterogeneity in continuous exercise volume, but do not show that nature connectedness reliably differentiates whether participants cross categorical exercise thresholds. Future research should measure perceived barriers, safety, social support, access to suitable activity spaces, exercise type, and socioeconomic factors directly, and test whether they account for the observed gender difference.

### 4.4 Implications for public mental health and health promotion

The Research Topic emphasizes supportive urban and residential environments, community resources, and inequalities in who is able to benefit from social and environmental change. The present findings fit this upstream perspective by linking perceived greenness and nature connectedness with physical exercise, a behavior with documented relevance to university students' mental health (1, 2). At the same time, the study does not demonstrate a mental-health effect because no mental-health outcome was included. The study's relevance to public mental health is therefore through the upstream determinants framework: understanding the environmental and psychosocial correlates of a health-promoting behavior contributes to the evidence base for upstream public mental health strategies, even when mental health outcomes themselves are not measured. A cautious implication is that public mental-health and university health-promotion strategies may benefit from considering environmental usability, opportunities for outdoor activity, and students' engagement with nature alongside sports facilities, curricula, and behavioral supports. Any implication that greater greenness or exercise would improve mental health is based on external evidence (1, 2) rather than a result of this study. Environmental exposures such as extreme heat have been directly linked to depressive symptoms in adolescents (9), underscoring the importance of understanding environmental pathways to mental health outcomes. Evidence that physical activity contributes to positive psychological functioning, including through moderated mediation pathways (37), further supports the value of supporting activity-friendly campus environments.

Research comparing outdoor and indoor exercise suggests that outdoor exercise often feels more enjoyable when intensity is matched, although physiological and longer-term behavioral advantages remain inconsistent (47, 48). Future university-based studies could jointly measure exercise volume, location of activity, nature connectedness, and mental-health outcomes. Such designs would allow direct testing of whether greener and more supportive environments facilitate sustained physical activity, whether physical activity contributes to subsequent mental-health outcomes, and whether these pathways differ by gender or socioeconomic position. This would more directly address questions of benefit and equity than the current cross-sectional design can do.

### 4.5 Limitations and future directions

Several limitations should be considered. First, the cross-sectional design precludes temporal or causal inference, and the statistical indirect effect should not be treated as causal mediation (45, 46). Cross-sectional mediation estimates can be systematically biased relative to longitudinal processes, particularly when variables refer to different time periods. Future studies should use at least three measurement waves or capitalize on natural experiments such as campus greening projects or residential relocation. Second, mental health was not included as an outcome in the present analysis. The paper therefore addresses environmental and behavioral resources relevant to public mental health, not mental-health status itself. Any implication that greater greenness or exercise would improve mental health is based on external evidence rather than a result of this study (1, 2).

Third, the environmental and behavioral time windows were not aligned: perceived greenness referred to the place lived for the longest period (potentially spanning many years), whereas exercise referred to the previous month. These two measures may therefore not refer to the same residential environment or the same time period, and the observed associations should be interpreted as concurrent statistical associations rather than temporally ordered effects. Future work should assess current campus and residential greenness concurrently with physical activity, combining subjective ratings with NDVI, street-view greenness, GPS trajectories, and wearable activity measures. Fourth, nature connectedness was assessed using the single-item INS measure. Although efficient, it does not separate cognitive, affective, and experiential dimensions (23–26). Fifth, PARS-3 is self-reported and may be affected by recall and social-desirability biases and does not identify where exercise occurred. Because the measure does not distinguish between indoor and outdoor exercise, we cannot evaluate whether the observed associations are specifically driven by “green exercise” (physical activity undertaken in natural settings). It is possible that nature connectedness is more strongly associated with outdoor than indoor activity, but the present data cannot test this distinction. Future studies should record exercise location and type to test whether nature connectedness is more strongly associated with outdoor activity and whether green exercise specifically mediates the environment–health relationship.

Sixth, the data came from a non-probability online cross-sectional sample, with a high proportion of first-year students (64.4%), and should not be used to estimate population prevalence for all Chinese university students. The archived project materials did not retain a site-by-site recruitment log, response rate, or case-level exclusion log, limiting our ability to evaluate the sample's representativeness across universities, regions, disciplines, or institutional contexts. The large sample also increases statistical power for small associations; the very small correlation between perceived greenness and exercise (*r* = 0.068) should therefore be interpreted in terms of magnitude as well as statistical significance. Seventh, the gender variable in the cleaned dataset included only male and female categories, limiting generalizability to gender-diverse populations and preventing a more intersectional equity analysis. Finally, potentially important factors—including exercise self-efficacy, peer support, academic workload, weather, facility quality, safety, and specific activity type—were not included (5–7), leaving scope for residual confounding. Reporting of this cross-sectional study follows the STROBE guidelines (49).

### 4.6 Conclusion

In this cross-sectional sample of 3,418 university students, perceived greenness of the long-term residential environment was weakly positively associated with exercise during the previous month. Perceived greenness showed a more robust positive association with nature connectedness, which was in turn associated with exercise, producing a significant concurrent statistical indirect association. The nature connectedness–exercise association and the conditional statistical indirect association were stronger in men. Findings were directionally similar across several continuous exercise scores, whereas the gender interaction was not significant after exercise was categorized. The exposure and outcome variables referred to different time windows (long-term residence vs. previous month), and the cross-sectional design does not establish temporal ordering. Viewed from an upstream public mental health perspective, the findings identify modest statistical links among a supportive environmental perception, a psychosocial resource, and a mental-health-relevant health behavior, while also indicating gender heterogeneity in these associations. They do not establish a causal mediation process or a direct effect on mental health, and the observed effect sizes were small.

## Data Availability

The raw data supporting the conclusions of this article will be made available by the authors, without undue reservation.
